# LaMYC7, a positive regulator of linalool and caryophyllene biosynthesis, confers plant resistance to *Pseudomonas syringae*

**DOI:** 10.1093/hr/uhae044

**Published:** 2024-02-06

**Authors:** Yanmei Dong, Ziling Wei, Wenying Zhang, Jingrui Li, Meixian Han, Hongtong Bai, Hui Li, Lei Shi

**Affiliations:** State Key Laboratory of Plant Diversity and Specialty Crops, Institute of Botany, Chinese Academy of Sciences, No.20 Nanxincun, Xiangshan, Beijing 100093, China; China National Botanical Garden, Beijing 100093, China; State Key Laboratory of Plant Diversity and Specialty Crops, Institute of Botany, Chinese Academy of Sciences, No.20 Nanxincun, Xiangshan, Beijing 100093, China; China National Botanical Garden, Beijing 100093, China; University of Chinese Academy of Sciences, Beijing 100049, China; State Key Laboratory of Plant Diversity and Specialty Crops, Institute of Botany, Chinese Academy of Sciences, No.20 Nanxincun, Xiangshan, Beijing 100093, China; China National Botanical Garden, Beijing 100093, China; University of Chinese Academy of Sciences, Beijing 100049, China; State Key Laboratory of Plant Diversity and Specialty Crops, Institute of Botany, Chinese Academy of Sciences, No.20 Nanxincun, Xiangshan, Beijing 100093, China; China National Botanical Garden, Beijing 100093, China; State Key Laboratory of Plant Diversity and Specialty Crops, Institute of Botany, Chinese Academy of Sciences, No.20 Nanxincun, Xiangshan, Beijing 100093, China; China National Botanical Garden, Beijing 100093, China; University of Chinese Academy of Sciences, Beijing 100049, China; State Key Laboratory of Plant Diversity and Specialty Crops, Institute of Botany, Chinese Academy of Sciences, No.20 Nanxincun, Xiangshan, Beijing 100093, China; China National Botanical Garden, Beijing 100093, China; State Key Laboratory of Plant Diversity and Specialty Crops, Institute of Botany, Chinese Academy of Sciences, No.20 Nanxincun, Xiangshan, Beijing 100093, China; China National Botanical Garden, Beijing 100093, China; State Key Laboratory of Plant Diversity and Specialty Crops, Institute of Botany, Chinese Academy of Sciences, No.20 Nanxincun, Xiangshan, Beijing 100093, China; China National Botanical Garden, Beijing 100093, China

## Abstract

Linalool and caryophyllene are the main monoterpene and sesquiterpene compounds in lavender; however, the genes regulating their biosynthesis still remain many unknowns. Here, we identified LaMYC7, a positive regulator of linalool and caryophyllene biosynthesis, confers plant resistance to *Pseudomonas syringae*. *LaMYC7* was highly expressed in glandular trichomes, and *LaMYC7* overexpression could significantly increase the linalool and caryophyllene contents and reduce susceptibility to *P. syringae* in *Nicotiana*. In addition, the linalool possessed antimicrobial activity against *P. syringae* growth and acted dose-dependently. Further analysis demonstrated that LaMYC7 directly bound to the promoter region of *LaTPS76*, which encodes the terpene synthase (TPS) for caryophyllene biosynthesis, and that *LaTPS76* was highly expressed in glandular trichomes. Notably, the *LaMYC7* promoter contained hormone and stress-responsive regulatory elements and responded to various treatments, including ultraviolet, low temperature, salt, drought, methyl jasmonate, and *P. syringae* infection treatments. Under these treatments, the changes in the linalool and caryophyllene contents were similar to those in *LaMYC7* transcript abundance. Based on the results, LaMYC7 could respond to *P. syringae* infection in addition to being involved in linalool and caryophyllene biosynthesis. Thus, the MYC transcription factor gene *LaMYC7* can be used in the breeding of high-yielding linalool and caryophyllene lavender varieties with pathogen resistance.

## Introduction

During plants' lives, they experience various environmental pressures, including biotic stressors (e.g. pathogens) and abiotic stressors (e.g. cold) [1, 2]. Plants have developed various defense systems against these stressors in order to survive and produce the next generation [3]. Among the several biotic stressors, pathogens pose the greatest risk to plant growth, development, and yield. *Pseudomonas syringae*, a gram-negative pathogenic bacterium, affects plants worldwide [4, 5]. Volatile terpenoids mount an effective defense in response to multiple stresses [6]. Volatile terpenoids, including monoterpenoids (e.g. linalool, pinene, myrcene, and linalyl acetate) and sesquiterpenoids (e.g. caryophyllene, farnesene, and germacrene), are the most common classes of volatile plant terpenoids. A high prevalence of linalool and caryophyllene is found in the plant kingdom, in general, and the Lamiaceae family, in particular [7, 8].

Linalool (C_10_H_18_O), an acyclic monoterpenoid, has ecological functions, such as serving as an attractant for both pollinators [9, 10] and predators [11], and acts as an antiherbivore defense to protect plants from damage [12, 13]. The compound is widely used in the pharmaceutical, cosmetic, food, and cleaning-product industries because of its pleasant scent, antibacterial properties, and sedative effects, among other properties [14–19]. Caryophyllene (C_15_H_24_; β-caryophyllene) is a bicyclic sesquiterpenoid with seemingly innumerable biological properties and commercial applications. In plants, β-caryophyllene contributes to lateral root formation, increases pathogen resistance, and provides plant resistance by jasmonic acid (JA) [20–22]. It is used as a fragrance or flavor compound in the cosmetics and food industries. Pharmacological studies have shown that β-caryophyllene has local anesthetic and anti-inflammatory effects, is used to treat depression and general anxiety, and can repel insects as well [23, 24].

The terpenoid biosynthetic pathway in plants has received a great deal of attention. Typically, geranyl diphosphate (GPP) and farnesyl diphosphate (FPP) are synthesized by the 2-C-methyl-d-erythritol 4-phosphate (MEP) pathway in plastids and the mevalonate pathway (MVA) in the cytoplasm, respectively. Different terpene synthases (TPSs) convert these compounds into monoterpenoids or sesquiterpenoids [25–27]. However, studies on the transcriptional regulation of volatile terpenoids are fewer than those on volatile terpenoid biosynthesis.

Transcription factors (TFs) control transcription or the simultaneous expression of several genes by binding to certain DNA sequences. Thus, TFs are considered the best targets for pathway engineering [28]. *MYC* genes play a pivotal role in secondary metabolite accumulation and are critical transcriptional activators that respond to JA signaling [29]. In *Arabidopsis*, AtMYC2 controls the transcript abundance of *AtTPS11* and *AtTPS21* to regulate caryophyllene biosynthesis [30, 31], and in the presence of JA, caryophyllene also equips plants with the ability to resist *P. syringae* pv. *tomato* (*Pst*) DC3000 [22]. However, the transcriptional regulation of volatile terpenoid remains elusive [32, 33]. The model plant, *Arabidopsis thaliana* has no glandular trichomes (GTs), which produce and accumulate terpenoids, and its terpenoid species are few. In contrast, lavender (*Lavandula angustifolia*), with more than 75 volatile terpenoids [34, 35], can serve as a model plant to study terpenoid regulation because a chromosome-based ‘Jingxun 2’ lavender genome has already been published [36].

In this study, by using RNA-sequencing, transgenic technology, solid-phase micro extraction coupled with gas chromatography–mass spectrometry (SPME-GC–MS), and quantitative reverse transcription polymerase chain reaction (qRT-PCR), enzyme activity, yeast one-hybrid (Y1H), and dual-luciferase (dual-LUC) assays, we comprehensively analyzed the expression of *LaMYC7* and its regulatory role in terpenoid synthesis in order to reveal its functions in protecting plants to cope with adversity, especially against *P. syringae* infection*.* Our findings not only serve as a basis for understanding how volatile terpenoid biosynthesis is controlled in lavender but also open the door to deciphering the transcriptional regulation of volatile terpenoids, as well as suggest that *LaMYC7* is a candidate gene for developing high-yielding and pathogen-resistant lavender plants.

**Figure 1:**
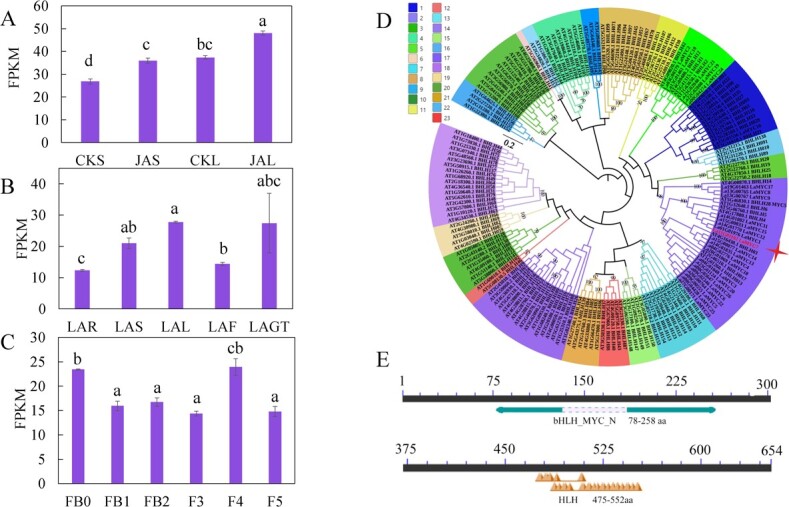
LaMYC7 characteristics. (A) Transcriptional alterations of *LaMYC7* from sepal or leaf with methyl jasmonate treatment (CKS, JAS, CKL, and JAL: CK, control; JA, methyl jasmonate treatment; S, sepal; L, leaf). (B) *LaMYC7* expression levels in *L. angustifolia* tissues (LAR, root; LAS, stem; LAL, leaf; LAF, flower; LAGT, glandular trichome). (C) Transcription abundance of *LaMYC7* at different stages (FB0, FB1, FB2, F3, F4, and F5; ‘F’ means flower, ‘FB’ means flower bud and ‘1–5’ five degrees of maturity). (D) Evolutionary tree analysis of LaMYC7 and AtMYC TFs. The method of neighbor-joining was used on MEGA7.0 to build the evolutionary tree, and 1000 replications of the bootstrap method were used to calculate the bootstrap values. (E) The NCBI (https://www.ncbi.nlm.nih.gov/Structure/cdd/wrpsb.cgi) was used to analyzed conserved domains. The numbers displayed are the average of at least three replicates (mean ± SD). The top of each bar represents standard errors, and bars annotated with different letters were significantly different according to Fisher’s LSD test (*P* < 0.05) after ANOVA.

## Results

### LaMYC7 isolation and bioinformatics analysis

Based on *L. angustifolia* genomic data (PRJNA642976), twenty-six MYCs were obtained using the hidden Markov model with PF14215 and PF00010 as queries (Supplementary Data Table S1), and the transcript abundance of the MYC gene *LaMYC7* increased after methyl jasmonate (MeJA) treatment (Fig. 1A). Compared with other tissues, the transcript abundance of *LaMYC7* was noticeably higher in leaf and GT, and gene expression was higher during FB0 and F4 flower development (Fig. 1B, C). The *LaMYC7* coding DNA sequence (CDS) was 1965 bp, encoding 654 amino acids (aas) (Fig. 1D, E). According to our bioinformatics study, LaMYC7 had a basic helix–loop–helix (bHLH)-MYC sequence (78–258 aa) and DNA-binding domain (475–552 aa) (Fig. 1E). The physicochemical properties of LaMYC7 were analyzed using ExPASy, and the isoelectric point and molecular weight of the protein were 5.31 and 71.25 kDa, respectively. According to the AtbHLH classification, LaMYC7 was clearly in the subfamily 2 or subgroup III (d + e) (Fig. 1D).

### Subcellular localization and transactivation activity of LaMYC7

**Figure 2:**
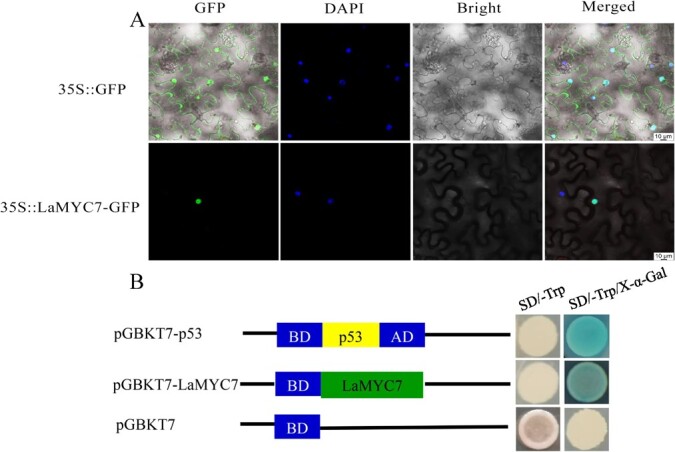
Analysis of LaMYC7 protein. (A) Subcellular localization used tobacco leaves. 35S::GPP was empty vector. 35S::LaMYC7-GFP was full length CDS of LaMYC7 recombined into the pCAMBIA2300 vector. The transformed tobacco leaves were then stained with 10 g/ml DAPI in order to be visualized. (B) Yeast AH109 cells with the positive control pGBKT7-p53, the recombined pGBKT7-LaMYC7, and the negative control pGBKT7 are shown in the top, middle, and bottom panels, respectively.

Subcellular localization of LaMYC7 in *Nicotiana* (*Nicotiana benthamiana*) leaves was determined by a transient expression test. 35S:LaMYC7-GFP was found exclusively in the nucleus of plant cells, while the empty vector (35S::GFP) was located in the nucleus and cytoplasm (Fig. 2A), indicating that LaMYC7 is localized in the nucleus.

The transactivation activity of LaMYC7 was evaluated using AH109 yeast cells and the pGBKT7 vector. AH109 cells transformed with each vector were grown on SD/−Trp medium. AH109 cells with the negative control vector (pGBKT7) did not appear blue, whereas AH109 cells with the recombinant pGBKT7-LaMYC7 vector or positive control vector (pGBKT7-p53) turned blue on SD/−Trp/X-α-Gal medium (Fig. 2B), indicating that LaMYC7 has transactivation activity.

### *LaMYC7* overexpression in *Nicotiana* increases volatile terpenoid biosynthesis

**Figure 3:**
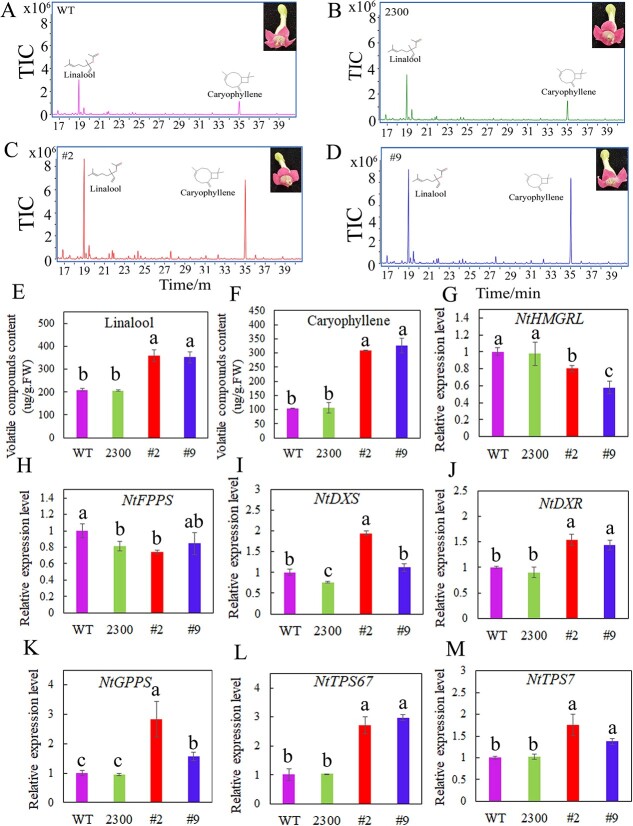
Analysis of the *LaMYC7*-overexpressing in tobacco. Wild-type (WT) plants transformed with the empty vector pCAMBIA2300 (2300) and *LaMYC7*-overexpressing plants with 35S::LaMYC7-GFP (#2, #9). (A-D) GC trace of caryophyllene and linalool. The peak area was indicated by the number on the peak. (E) Linalool content. (F) Caryophyllene content. (G-M) Relative expression levels of *NtHMGRL*, *NtFPPS*, *NtDXS*, *NtDXR*, *NtGPPS*, *NtTPS76* and *NtTPS7*. By comparing the products to substances in the NIST14 collection and reference standards, the compounds were identified. The numbers displayed are the average of at least three replicates (mean ± SD). Following an ANOVA, Fisher’s LSD test revealed that bars labeled with various letters were significantly different (*P* < 0.05), as seen by the vertical lines at the top of each bar indicating standard errors.

To assess the role of LaMYC7 in volatile terpenoid biosynthesis, *LaMYC7* was overexpressed in *Nicotiana* and the T2 generation of transgenic lines #2 and #9 was selected for further research. Terpenoid content and gene expression levels were measured using SPME-GC–MS and qRT-PCR, respectively. The total volatile terpenoid, sesquiterpenoid, and monoterpenoid contents were significantly increased, which was in accordance with *LaMYC7* overexpression in *Arabidopsis* (Supplementary Data Fig. S1, 2). Notably, the linalool and caryophyllene levels were approximately 0.71- and 1.98-fold higher in *LaMYC7*-overexpressing lines #2 and #9, respectively, compared with control 2300 plants (Fig. 3A–F and Supplementary Data Fig. S3). In addition, the relative expression levels of *NtHMGL* and *NtFPPS*, key enzymes that control sesquiterpene biosynthesis, were decreased in the flowers of *LaMYC7*-overexpressing *Nicotiana* lines #2 and #9 compared with control 2300 plants (Fig. 3G, H). However, the relative expression levels of *NtDXS*, *NtDXR*, and *NtGPPS*, key enzymes that control monoterpene biosynthesis, were significantly increased in *LaMYC7*-overexpressing *Nicotiana* lines #2 and #9 compared with control 2300 plants (Fig. 3I–K). Moreover, the expression levels of linalool synthase (*NtTPS67*) and caryophyllene synthase (*NtTPS7*) were consistent with linalool and caryophyllene accumulation, which was significantly increased in *LaMYC7*-overexpressing *Nicotiana* lines #2 and #9 compared with control plants (Fig. 3L, M). However, *LaMYC7* overexpression in *Arabidopsis* could increase the linalool content but not significantly, while the caryophyllene content significantly increased (>7-fold) (Supplementary Data Fig. S4A-F and S5). The relative gene expression levels of key enzymes that control terpenoid biosynthesis and some TPSs showed that *LaMYC7* overexpression in *Arabidopsis* could significantly increase the expression levels of *AtHMGR1*, *AtFPPS1*, *AtDXR*, *AtGPPS*, and caryophyllene synthase (*AtTPS21*) (Supplementary Data Fig. S4G–M).

In transgenic *Nicotiana*, the zeatin riboside (ZR), indole acetic acid (IAA), and JA levels were significantly decreased compared with control plants. However, the changes in the gibberellin (GA_3_) content were not significant, and the abscisic acid (ABA) content significantly increased (Supplementary Data Fig. S6). *LaMYC7* overexpression had no effect on plant height or total anthocyanin content (TAC) in transgenic *Nicotiana*, but chlorophyll and carotenoid biosynthesis decreased (Supplementary Data Fig. S7).

### *LaMYC7* overexpression confers *Nicotiana* with resistance to *P. Syringae*

**Figure 4:**
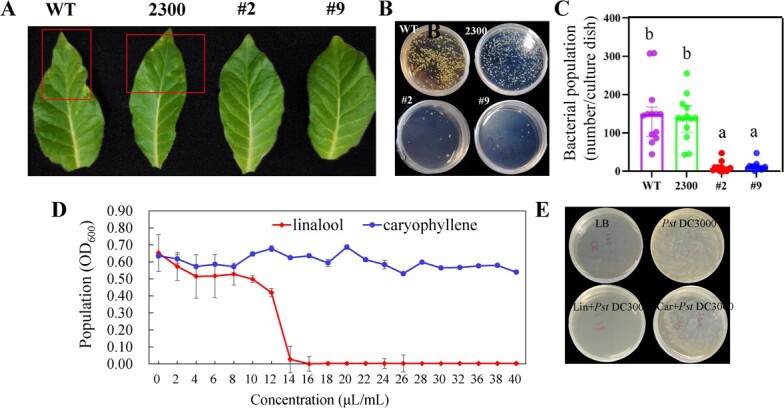
Analysis of the potential biological role of LaMYC7 for *P. syringae*. A, *P. syringae* infection of WT, 2300, and *LaMYC7*-overexpressing transgenic lines for 5 d for phenotype analysis. B, C, Bacterial population at 5 d in WT, 2300 and *LaMYC7*-overexpressing transgenic lines (#2, #9). D, E, Antibacterial activity of linalool and caryophyllene against *Pst* DC3000. LB, Empty lysogeny broth; *Pst* DC3000, 150 μl *Pst* DC3000 were dissolved in lysogeny broth medium; Lin + *Pst* DC3000, 150 μl *Pst* DC3000 were dissolved in lysogeny broth medium containing 18 μl∙ml^−1^ linalool. Car + *Pst* DC3000, 150 μl *Pst* DC3000 were dissolved in lysogeny broth medium containing 18 μl∙ml^−1^ caryophyllene. The numbers displayed are the average of at least three replicates (mean ± SD). Following an ANOVA, Fisher’s LSD test revealed that bars labeled with various letters were substantially different (*P* < 0.05), as seen by the vertical lines at the top of each bar indicating standard errors.

The phenotypes of *LaMYC7*-overexpressing lines (i.e. #2 and #9) and control plants (i.e. wild-type (WT) and 2300) inoculated with *Pst* DC3000 were studied after 5 days to elucidate the potential biological role of LaMYC7 in plant disease prevention. Necrotic spots were found in WT and empty vector plants, while *LaMYC7*-overexpressing lines grew normally (Fig. 4A). In addition, bacterial growth on plants was assessed using *Pst* DC3000. The outcomes of the statistical analysis revealed that the bacterial population was dramatically decreased in *LaMYC7*-overexpressing lines compared with control plants (Fig. 4B, C). Furthermore, the antimicrobial activity of linalool and caryophyllene against *Pst* DC3000 growth was evaluated. Linalool inhibited *Pst* DC3000 growth in a dose-dependent manner and showed strong antimicrobial activity regardless of *Pst* DC3000, while caryophyllene did not show any antibacterial activity regardless of *Pst* DC3000 at a concentration of 40 μl/ml (Fig. 4D, E).

### Isolation and characteristics of caryophyllene synthase

**Figure 5:**
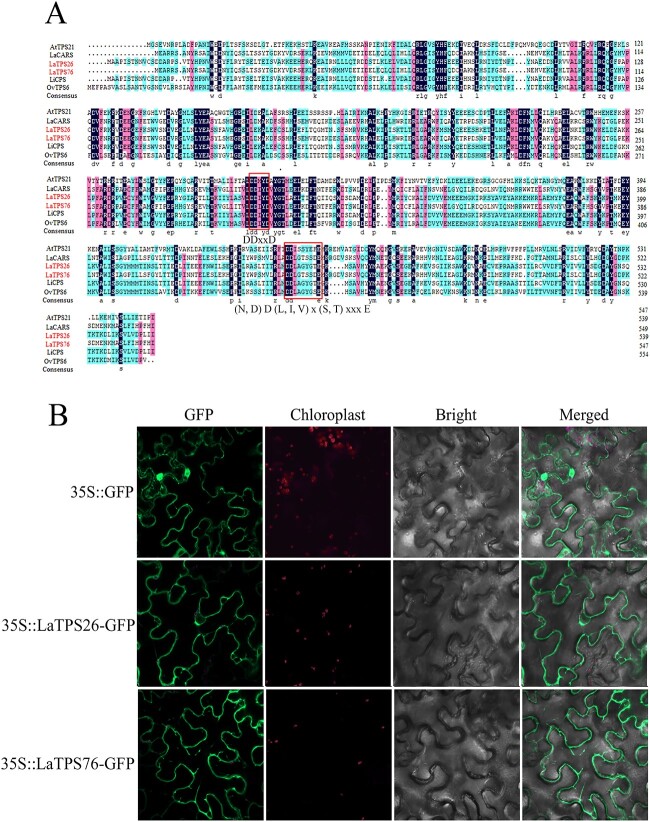
Sequence analysis and subcellular localization of LaTPS26 and LaTPS76. A, Multiple alignment of TPSs. B, Subcellular localization of LaTPS26 and LaTPS76 in *Nicotiana* leaves.

Based on genomic data (PRJNA642976), 100 TPSs were previously found in *L. angustifolia* [34]. The *TPS* genes *LaTPS26* (La05G01453) and *LaTPS76* (La22G02785) were present in the turquoise module along with *LaMYC7* in a weighted correlation network analysis (WGCNA) (unpublished). In the gene expression profiling of various tissues, *LaTPS26* and *LaTPS76* had the highest levels of expression in GTs, and the expression level of *LaTPS76* was significantly greater than that of *LaTPS26* (Supplementary Data Fig. S8). The open reading frames of *LaTPS26* and *LaTPS76* encoded 549- and 540-aa proteins, respectively. The protein sequences of LaTPS26 and LaTPS76 contained DDxxD and (N, D) D (L, I, V) x (S, T) xxx E motifs, which are the typical TPS domains (Fig. 5A). Protein subcellular localization prediction (WoLF PSORT; https://wolfpsort.hgc.jp/) showed that LaTPS26 and LaTPS76 were localized in the cytoplasm. To confirm the subcellular localization of LaTPS26 and LaTPS76, LaTPS26-GFP and LaTPS76-GFP fusion proteins were produced and transformed into *Agrobacterium tumefaciens* GV3101. 35S::GFPs were identified in both cytoplasm and nucleus, but LaTPS26-GFP and LaTPS76-GFP were identified exclusively in the cytoplasm (Fig. 5B), showing that LaTPS26 and LaTPS76 localize in the cytoplasm.

**Figure 6:**
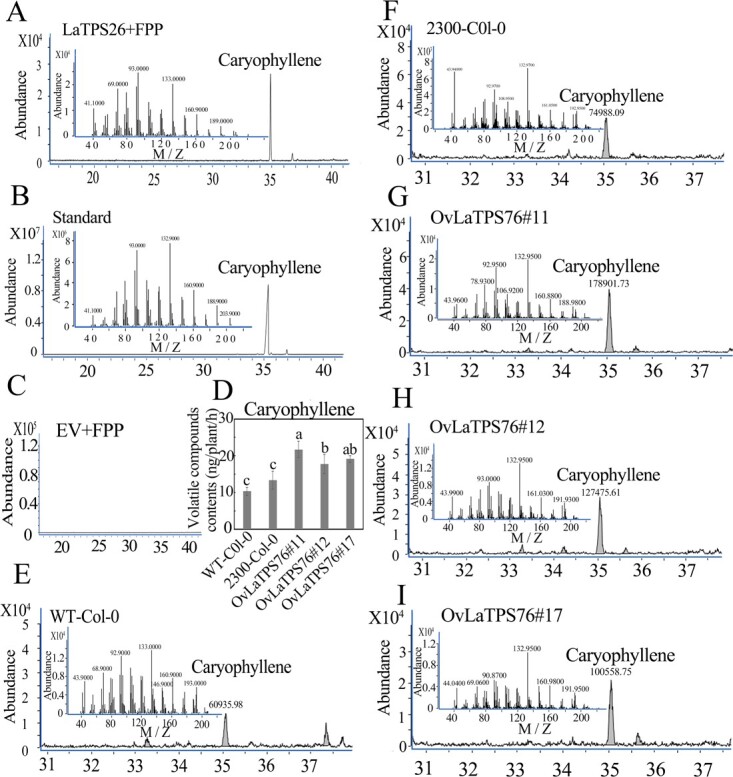
Functional analysis of LaTPS26 and LaTPS76. A, C, GC trace of products from the heterologously expressed proteins of empty vector (pGEX-4T1) and pGEX-4T1-LaTPS26 using farnesyl diphosphate (FPP) as substrate. B, The GC–MS spectrum of the caryophyllene standard was utilized as a guide. D, Caryophyllene contents from *Arabidopsis* plants. E-I, GC trace of caryophyllene. Colombia wild-type (WT-Col-0) plants transformed with the empty vector pCAMBIA2300 (2300) and the LaMYC7-overexpressing plants with 35S:LaMYC7-GFP (#11, #12, #17).

To ascertain the biological roles of LaTPS26 and LaTPS76, the enzyme activity of the LaTPS26 protein and *LaTPS76*-overexpressing transgenic *Arabidopsis* were characterized. Enzyme activity assays demonstrated that LaTPS26 can convert FPP to caryophyllene (Fig. 6A–C). In addition, the caryophyllene content significantly increased in *LaTPS76*-overexpressing transgenic plants compared with control plants (Fig. 6D–I). In addition, we found four homologous genes of linalool synthase (based on the published ‘Jingxun 2’ lavender genome) which were highly expressed in GTs. However, among these four genes, the highest FPKM value was 235 (Supplementary Data Fig. S9).

### LaMYC7 directly binds to the *LaTPS76* promoter

**Figure 7:**
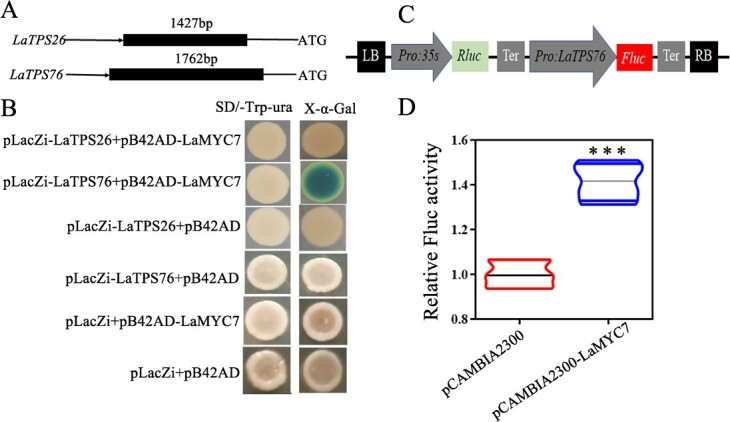
LaMYC7 binds to the LaTPS76 promoter and activates its transcription. A, promoter. B, Color reaction. SD/−Trp/–Ura selection media containing 80 mg∙l^−1^ X-α-Gal. C, Schematic of the dual-luciferase system used for promoter activity assay. LB, left border; RB, right border; Ter, terminator. D, Relative luciferase activities (LUC/REN ratio) for co-expressed LaTPS76 pro::LUC + 35S:: LaMYC7 and LaTPS76 pro::LUC + pCAMBIA2300. Values represent mean ± standard deviation (n = 4). The value of the negative control was used as the reference and set to 1, error bars denote standard deviations, and asterisks indicate a statistically significant difference (two-sided Student’s *t*-test; ^***^P < 0.001).

Because LaMYC7, LaTPS27, and LaTPS76 were related to caryophyllene biosynthesis, the regulatory connection between LaMYC7 and *LaTPS26/LaTPS76* was examined. The promoters were also inserted into pLacZi vectors (Fig. 7A). After being co-transformed with the pB42AD-MYC7 or empty pB42AD vector, the Y1H assay results showed that all co-transformed cells survived on SD-Trp/Ura medium, while only co-transformed cells with pLacZi-TPS76 and pB42AD-MYC7 turned blue on SD-Trp/Ura/X-gal medium (Fig. 7B). This implied that LaMYC7 could directly bind to the *LaTPS76* promoter but was unable to bind to the *LaTPS26* promoter. Furthermore, the *LaTPS76* promoter was inserted into the dual-LUC reporter plasmid containing firefly luciferase (FLuc) and Renilla luciferase (RLuc) reporter genes (Fig. 7C). LaMYC7 could also significantly activate the *LaTPS76* promoter (Fig. 7D).

### Analysis of the *LaMYC7* promoter sequence and stress response

**Figure 8:**
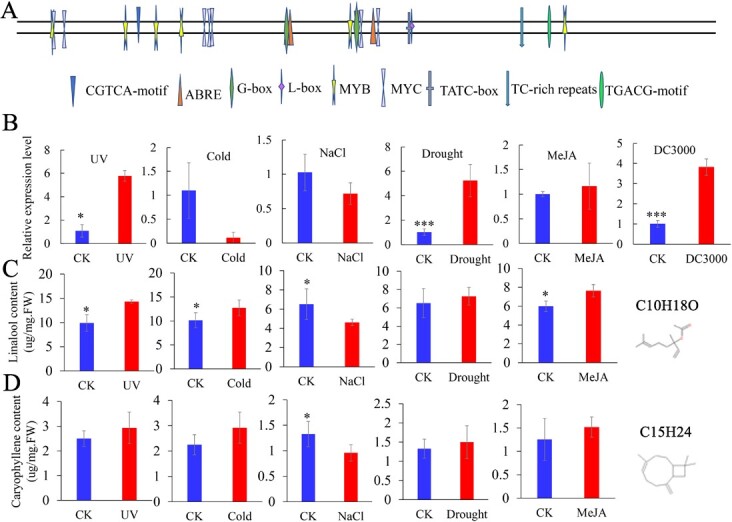
Analysis of the promoter and expression level of *LaMYC7* and the changes of linalool and caryophyllene contents under various stresses. A, Analysis of the *LaMYC7* promoter sequence. The PlantCARE database was utilized to find potential cis-acting regulatory elements. B, Relative expression of *LaMYC7* was measured using qPCR. C, Linalool content under various stresses. D, Caryophyllene content under various stresses. The number displayed are the average of at least three replicates (mean ± SD). Following an ANOVA, *t* test revealed that bars labeled with ^*^ (*p* < 0.05), ^**^ (*p* < 0.01), ^***^ (*p* < 0.001) were significantly different, as seen by the vertical lines at the top of each bar indicating standard errors.

The *LaMYC7* promoter sequence, a sequence 2000-bp upstream from the *LaMYC7* translation initiation site, was evaluated by PlantCARE software (Supplementary Data Table S2). Four ABA-responsive elements were located at +776, −1039, +777, and +1040 bp. One TATC-box, which responds to GA, was found at +1128 bp. One TC-rich repeat element, which participates in stress response and defense, was found at +1455 bp. Two MeJA-responsiveness elements were found at −1666 and +1666 bp (Fig. 8A and Supplementary Data Table S2). Furthermore, LaMYC7 confers plant tolerance to drought stress in *LaMYC7*-overexpressing plants (Supplementary Data Fig. S10).

*LaMYC7* expression levels under different adversity treatments were measured using qRT-PCR. In lavender, ultraviolet (UV), drought, MeJA, and *Pst* DC3000 infection treatments elevated *LaMYC7* expression by 5-, 4-, 0.2-, and 3-fold, respectively, while cold and salt treatments dramatically downregulated it by 0.8- and 0.3-fold, respectively (Fig. 8B). In addition, the changes in the linalool and caryophyllene contents were similar to those in *LaMYC7* transcript abundance in UV, salt, drought and MeJA treatments (Fig. 8C, D).

## Discussion

Linalool and caryophyllene have multiple ecological functions, including chemical signals for plant–pollinator interactions, antiherbivory, and pathogen resistance [9–11, 22]. In addition to their ecological functions, these compounds find applications in the pharmaceutical, cosmetic, and food industries [14–16, 23, 24]. Linalool and caryophyllene are formed by TPSs using GPP and FPP as substrates [37, 38]; however, the transcriptional regulation mechanism underlying their biosynthesis is unclear. In the model plant *Arabidopsis*, AtMYC2 participated in regulating caryophyllene biosynthesis by binding to *TPS21* and *TPS11* promoters to regulate their expression [30]. *LaMYC4* (now called *LaMYC17*) overexpression increased the caryophyllene content in tobacco [39], whereas the caryophyllene content increased in *SlMYC1*-downregulated lines, and FhMYC2 interacted with FhMYB21 to regulate the expression of *TPS1*, which produces linalool [40, 41]. We extensively searched the lavender genome and identified 26 putative MYC TFs (LaMYC1–26) containing the bHLH-MYC_N and bHLH domains. Gene expression analysis indicated that *LaMYC7* was differentially expressed after MeJA treatment (upregulation) and highly expressed in GTs (Fig. 1A, B). Therefore, *LaMYC7* was further analyzed.

*LaMYC7* overexpression resulted in increased linalool and caryophyllene contents in *Nicotiana* (Fig. 3A-F and Supplementary Data Fig. S4A-F). In addition, the transcript levels of *NtTPS67* and *NtTPS7*, the structural genes of the linalool and caryophyllene biosynthetic pathway, were significantly increased in *LaMYC7*-overexpressing lines (Fig. 3L, M). The transcript levels of the MEP pathway key genes *NtDXS* and *NtGPPS* were significantly increased, while the transcript level of *NtHMGRL*, a key gene of the MVA pathway, was significantly decreased (Fig. 3G–K). These results indicated that LaMYC7 regulated linalool and caryophyllene biosynthesis and influenced carbon flow in the MEP pathway. Notably, *LaMYC7* overexpression resulted in enhancing the transcript levels of some early pathway genes (e.g. *AtHMGR1*, *AtFPPS1*, *AtDXS*, *AtDXR*, and *AtGPPS*) in *Arabidopsis* (Supplementary Data Fig. S4G-K). The caryophyllene content and caryophyllene synthase transcript level were significantly increased, while the linalool content and linalool synthase transcript level did not change significantly (Supplementary Data Fig. S4A–F, L, M). Volatile terpenoids also need specialized storage structures to prevent autocytotoxicity; for example, monoterpenoids are harmful to unspecialized plant cells and must be sequestered [42, 43]. Because *Arabidopsis* lacks GTs, it has fewer volatile compounds, especially monoterpenoids such as linalool. These may be the reasons for the difference in the results of *LaMYC7* overexpression in plants with and without GTs.

In addition to the role of LaMYC7 in linalool and caryophyllene biosynthesis, its role in regulating stress was investigated. Plants are subjected to various stresses during their entire lives, and they have thus developed several defense mechanisms to withstand these stresses [44–46]. The *LaMYC7* promoter analysis results suggested that LaMYC7 plays an important role in environmental adaptation. Furthermore, the results of UV, MeJA, drought, low temperature, salt, and *P. syringae* infection treatments indicated that LaMYC7 can respond to multiple stresses (Fig. 8A, B). *LaMYC7* overexpression in *Nicotiana* could significantly increase resistance to *P. syringae* (Fig. 4A–C), which causes economically important plant diseases [4]. Caryophyllene has been shown to confer plant resistance to *P. syringae* infection through the JA signaling pathway [22]. However, *LaMYC7* overexpression in *Nicotiana* decreased JA levels (Supplementary Data Fig. S6). The negative feedback mechanism enables plants to achieve a dynamic balance to ensure normal growth. The antibacterial activity results showed strong antimicrobial activity irrespective of the presence of *P. syringae* at concentrations exceeding 16 μl/ml and dose-dependently; however, caryophyllene did not show any antibacterial activity regardless of *Pst* DC3000 at a concentration of 40 μl/ml (Fig. 4D, E). Therefore, *LaMYC7* overexpression conferred plant resistance to *P. syringae*, which may have been achieved by linalool.

The intricacy of terpenoid regulation is illustrated by the fact that TPSs frequently have numerous copies to assist complex metabolic processes [47]. The *TPS* genes *LaTPS26* (La05G01453) and *LaTPS76* (La22G02785) in this study were found in the turquoise module with *LaMYC7* by WGCNA (unpublished). According to the results of the gene expression profiling of various tissues, *LaTPS26* and *LaTPS76* had the highest levels of expression in GTs, and the expression level of *LaTPS76* was significantly greater than that of *LaTPS26* (20-fold) (Supplementary Data Fig. S8). Transgenic and in vitro enzyme activity analysis showed that both LaTPS26 and LaTPS76 can synthesize caryophyllene (Fig. 6). Notably, the results of the Y1H and LUC assays showed that LaMYC7 can only bind to the *LaTPS76* promoter, indicating that LaMYC7 regulates caryophyllene synthesis by binding to the *LaTPS76* promoter. In addition, the tissue expression analysis results showed that the four homologous genes of linalool synthase were highly expressed in GTs, FPKM of that were far lower than *LaTPS76* (Supplementary Data Fig. S9). However, in *L. angustifolia* essential oil, the content of linalool was more than 20-fold that of caryophyllene [48, 49]. Thus, the four homologous genes of linalool synthase may not be major-effect genes Fig. S9. Therefore, further research is needed to elucidate linalool regulation by LaMYC7.

## Conclusions

To our knowledge, LaMYC7, isolated from the lavender cultivar ‘Jingxun 2’in this study, is the first MYC TF shown to be a positive regulator of linalool and caryophyllene biosynthesis as well as a disease resistance-responsive MYC TF that actively regulates disease resistance. In addition, LaMYC7 bound to the promoter of LaTPS76, which produces caryophyllene. Furthermore, *LaMYC7* overexpression conferred plant resistance to *Pst* DC3000, while the JA level decreased. Linalool exhibited strong antibacterial activity against *Pst* DC3000 growth *in vitro*. Thus, we identified a novel MYC TF, LaMYC7, which has roles in linalool and caryophyllene biosynthesis as well as disease resistance in lavender.

## Materials and methods

### Plant materials and stress treatments

Lavender (*L. angustifolia* cultivar ‘Jingxun 2’), *Arabidopsis* (*A. thaliana* Col-0), and *Nicotiana* (*N. benthamiana* and *Nicotiana tabacum*) were used in this study. Cuttings were used to grow ‘Jingxun 2’ in potting soil. All plants were planted in the greenhouse of the Institute of Botany, Chinese Academy of Sciences (Beijing, China). UV, cold, salt (NaCl), drought, and MeJA treatment and *Pst* DC3000 inoculation as previously described [37]. The following acronyms were used for samples: root (R), stem (S), leaf (L), flower (F), sepal (S), glandular trichomes (GT), flower bud (FB). FB0, FB1, FB2, F3, F4, and F5 indicate the stages of flower growth [50].

### Subcellular localization and transactivating activity of LaMYC7

The primer pairs (Supplementary Data Table S4) used for *LaMYC7*, *LaTPS26*, and *LaTPS76* were based on their full-length CDSs obtained from the lavender genome (PRJNA642976) and the KpnI restriction site sequence of the pCAMBIA2300 vector. *LaMYC7*, *LaTPS26*, and *LaTPS76* were isolated from sepal cDNA using PCR. The PCR products were recombined into the empty vector pCAMBIA2300 to produce the recombinant vectors 35S::LaMYC7-GFP, 35S::LaTPS26-GFP, and 35S::LaTPS76-GFP. *A. tumefaciens* GV3101 was heat-shock transformed with the empty and vectors for subcellular localization analysis. As previously described [51], *N. benthamiana* leaves were also transformed with the recombinant vectors and the empty vector 35S::GFP. After 3 days, subcellular localization in the leaves was analyzed under a confocal laser scanning microscope (Leica TCS SP5; Leica Microsystems, Mannheim, Germany).

For the transactivation activity assay, the LaMYC7 sequence was recombined into the pGBKT7 vector. The recombinant (pGBKT7-LaMYC7), positive control (pGBKT7-p53), and negative control (pGBKT7) vectors were transformed and expressed in AH109 yeast cells.

### Sequence analysis of LaMYC7

*Arabidopsis* bHLH protein (AtbHLH) sequences were acquired from TAIR database (http://www.arabidopsis.org). Two specific MYC domains PF14215 and PF00010 were used to query the *L. angustifolia* genome database (PRJNA642976). Based on the AtbHLH and LaMYC protein sequences, MEGA 7.0 software was used to construct a phylogenetic tree using the neighbor-joining method with 1000 replicates. Characteristics of the LaMYC7 protein sequence were determined by the Compute pI/Mw tool (ExPASy; https://web.expasy.org/compute_pi/). For the functional prediction analysis of *cis*-regulating elements, the promoter sequence (2000-bp upstream of the translation initiation site) of the *LaMYC7* gene was submitted to PlantCARE software (http://bioinformatics.psb.ugent.be/webtools/plantcare/html/).

### qRT-PCR analysis

qRT-PCR was performed on the Stratagene Mx3000P system (Agilent Technologies, Palo Alto, CA, USA). The PCR procedure and data analysis were completed as previously described [52].

### Plant transformation

Using the leaf disk method for *Nicotiana* [53] or the floral dip method for *Arabidopsis* [54], bacterial colonies bearing the 35S::LaMYC7-GFP vector were selected and transformed. Plants infiltrated with the empty vector were used as control. Transgenic T0 generation seeds were preliminarily screened using 50 g∙ml^−1^ kanamycin on half-strength Murashige and Skoog medium before PCR identification.

### TPS functional analysis

The full-length CDS of *LaTPS26* was recombined into the pGEX-4T1 vector to produce a recombinant plasmid (pGEX-4T1-LaTPS26). This plasmid was then introduced into *Escherichia coli* DH5α and sequenced to ensure proper insertion. The corrected plasmid (pGEX-4T1-LaTPS26) was introduced into the *E. coli* strain BL21 (DE3). Production and purification of heterologous proteins were performed as previously described [55]. The *in vitro* enzymatic assay for LaTPS26 activity was performed in headspace vials according to the description of Chen *et al*. [56] in a reaction mixture (500 μl) containing buffer (25 mM HEPES, pH 7.0, 100 mM KCl, 10 mM MgCl_2_, 10 mM MnCl_2_, 10% glycerol, and 10 mM DTT), purified protein (20–50 μg per reaction), and 10 μg FPP (Sigma–Aldrich, St. Louis, MI, USA). The reaction mixture was incubated at 30°C for 8 h. The products were analyzed by SPME-GC–MS.

*LaTPS76* isolated from sepal cDNA by PCR was recombined into the pCAMBIA2300 vector. *LaTPS76-*overexpressing transgenic *Arabidopsis* plants were obtained by *A. tumefaciens*-mediated transformation. Terpenoid levels were analyzed in the T3 generation. Sequences and primers of *LaTPS26* and *LaTPS76* are shown in Tables S3 and S4.

### Measurement of volatile terpenoid content

Volatile compounds emitted by *Arabidopsis* and *Nicotiana* were collected by SPME as previously described [39]. Samples were injected in the splitless mode. Products were identified based on retention times, electron ionization mass spectra from the NIST Mass Spectral Library (NIST-14.0), and information from the literature [34, 57].

### Yeast one-hybrid assay

Y1H assays were performed as previously described [58]. The *LaMYC7* CDS was ligated into the pB42AD vector. The 1427-bp *LaTPS26* promoter or the 1762-bp *LaTPS76* promoter was isolated and inserted into the pLacZi vector. The recombinant pLacZi-LaTPS26/pB42AD-LaMYC7, pLacZi-LaTPS76/pB42AD-LaMYC7, pLacZi-LaTPS26/pB42AD, pLacZi-LaTPS76/pB42AD, pLacZi/pB42AD-LaMYC7, or empty pLacZi/pB42AD vector was introduced into EGY48 yeast cells, which were then grown on SD/−Trp/-Ura selection medium for 72 h before being assayed for color development on the same medium with 40 mg∙L^−1^ 5-bromo-4-chloro-3-indolyl-α-d-galactopyranoside (X-α-Gal).

### Dual-luciferase (dual-LUC) assay in tobacco leaves

For the dual-LUC assays, the *LaTPS76* promoter was inserted into the pGreenII 0800-LUC vector as a reporter. The effector and reporter were transformed into *A. tumefaciens* strain GV3101 with the helper plasmid pSoup-19. *Agrobacterium* harboring the reporter vector was then co-infiltrated into *N. benthamiana* leaves with *Agrobacterium* carrying either the *LaMYC7*-overexpressing vector (pCAMBIA2300-*LaMYC7*) or the control vector (pCAMBIA2300) in a 1:1 ratio. After incubation at 22°C for 36 hours, the agro-infiltrated leaves were collected, and the FLuc activity was quantitatively analyzed using a dual-LUC assay kit (Yeasen Biotechnology, Shanghai). The analysis was performed using the GloMax 20/20 luminometer (E5311; Promega) according to the manufacturer's instructions. At least five measurements were performed for each assay.

### Measurement of TAC and endogenous hormone contents

Twelve plants from each sample were selected to assess plant height and TAC. TAC in 500 mg of *Nicotiana* flowers was measured as previously described [31]. The ZR, IAA, JA, GA_3_, and ABA levels were measured in *Nicotiana* leaves using ELISA. Hormones were isolated, purified, and quantified by ELISA as described by He [59] and Yang *et al*. [60].

### Assessment of pathogen and drought tolerance in transgenic plants

The stress resistance abilities of WT, empty vector (2300), and transgenic (#2 and #9) plants were investigated. To simulate drought stress, four-week-old potted *Nicotiana* plants were grown without water for 45 days in a greenhouse. Plants were given sufficient water to rehydrate, and they were observed and photographed the following day. For pathogen infection treatment, 8-week-old potted *Nicotiana* plants were inoculated with *Pst* DC3000. *Pst* DC3000 solution for inoculation was prepared by the method of Chen *et al*. [61]. The WT, 2300, #2, and #9 plants were sprayed with *Pst* DC3000 solution. Five days after *Pst* DC3000 inoculation, six leaves from a single plant were harvested and rinsed twice with sterile water. The leaf-infected regions were excised using a hole punch, and the disks were homogenized in Luria Broth containing rifampicin and then cultured on solid medium for 2 days at 28°C.

## Supplementary Material

Web_Material_uhae044

## Data Availability

The raw genome and transcriptome sequencing data reported in this paper have been deposited in the National Center for Biotechnology Information (NCBI) database under project number PRJNA642976. And the data and materials in the current study are available from the corresponding author on reasonable request.

## References

[1] Suzuki N , Rivero RM, Shulaev V. et al. Abiotic and biotic stress combinations. New Phytol. 2014;203:32–43 24720847 10.1111/nph.12797

[2] Chisholm ST , Coaker G, Day B. et al. Host-microbe interactions: shaping the evolution of the plant immune response. Cell. 2006;124:803–14 16497589 10.1016/j.cell.2006.02.008

[3] Atkinson NJ , Urwin PE. The interaction of plant biotic and abiotic stresses: from genes to the field. J Exp Bot. 2012;63:3523–43 22467407 10.1093/jxb/ers100

[4] Fan L , Wang T, Hua C. et al. A compendium of DNA-binding specificities of transcription factors in pseudomonas syringae. Nat Commun. 2020;11:4947 33009392 10.1038/s41467-020-18744-7 PMC7532196

[5] Zhao C , Liu W, Zhang YL. et al. Two transcription factors, AcREM14 and AcC3H1, enhance the resistance of kiwifruit *Actinidia chinensis* var. *chinensis* to *pseudomonas syringae* pv. Actinidiae. Hortic. Res. 2024;11:uhad242 38222821 10.1093/hr/uhad242 PMC10782502

[6] Jin JY , Zhao MY, Jing TT. et al. Volatile compound-mediated plant–plant interactions under stress with the tea plant as a model. Hortic Res. 2023;10:uhad143 37691961 10.1093/hr/uhad143 PMC10483893

[7] Mihajilov-Krstev T , Radnović D, Kitić D. et al. Chemical composition, antimicrobial, antioxidative and anticholinesterase activity of Satureja Montana L. ssp Montana essential oil. Open Life Sci. 2014;9:668–77

[8] Carrasco A , Martinez-Gutierrez R, Tomas V. et al. *Lavandula angustifolia* and *Lavandula latifolia* essential oils from Spain: aromatic profile and bioactivities. Planta Med. 2016;82:163–70 26441063 10.1055/s-0035-1558095

[9] Miyake T , Yamaoka R, Yahara T. Floral scents of hawkmoth-pollinated flowers in Japan. J Plant Res. 1998;111:199–205

[10] Raguso RA . More lessons from linalool: insights gained from a ubiquitous floral volatile. Curr Opin Plant Biol. 2016;32:31–6 27286000 10.1016/j.pbi.2016.05.007

[11] Kessler A , Baldwin IT. Defensive function of herbivore-induced plant volatile emissions in nature. Science. 2001;291:2141–4 11251117 10.1126/science.291.5511.2141

[12] Turlings TC , Loughrin JH, McCall PJ. et al. How caterpillar-damaged plants protect themselves by attracting parasitic wasps. Proc Natl Acad Sci U S A. 1995;92:4169–74 7753779 10.1073/pnas.92.10.4169 PMC41905

[13] Turlings TCJ , Tumlinson JH, Lewis WJ. Exploitation of herbivore-induced plant odors by host-seeking parasitic wasps. Science. 1990;250:1251–3 17829213 10.1126/science.250.4985.1251

[14] Lapczynski A , Letizia CS, Api AM. Addendum to fragrance material review on linalool. Food Chem Toxicol. 2008;46:S190–2 19097259 10.1016/j.fct.2008.06.087

[15] Aprotosoaie AC , Hăncianu M, Costache I-I. et al. Linalool: a review on a key odorant molecule with valuable biological properties: linalool: a key odorant molecule. Flavour & Fragrance J. 2014;29:193–219

[16] Ma J , Xu H, Wu J. et al. Linalool inhibits cigarette smoke-induced lung inflammation by inhibiting NF-κB activation. Int Immunopharmacol. 2015;29:708–13 26432179 10.1016/j.intimp.2015.09.005

[17] Herman A , Tambor K, Herman A. Linalool affects the antimicrobial efficacy of essential oils. Curr Microbiol. 2016;72:165–72 26553262 10.1007/s00284-015-0933-4

[18] Dos Santos ÉRQ , Maia CSF, Fontes Junior EA. et al. Linalool-rich essential oils from the Amazon display antidepressant-type effect in rodents. J Ethnopharmacol. 2018;212:43–9 29037915 10.1016/j.jep.2017.10.013

[19] Pereira I , Severino P, Santos AC. et al. Linalool bioactive properties and potential applicability in drug delivery systems. JCIS Open. 2018;171:566–78 10.1016/j.colsurfb.2018.08.001 30098535

[20] Ditengou FA , Müller A, Rosenkranz M. et al. Volatile signaling by sesquiterpenes from ectomycorrhizal fungi reprogrammes root architecture. Nat Commun. 2015;6:6279 25703994 10.1038/ncomms7279 PMC4346619

[21] Yamagiwa Y , Inagaki Y, Ichinose Y. et al. Talaromyces wortmannii FS2 emits β-caryphyllene, which promotes plant growth and induces resistance. J Gen Plant Pathol. 2011;77:336–41

[22] Frank L , Wenig M, Ghirardo A. et al. Isoprene and β-caryophyllene confer plant resistance via different plant internal signaling pathways. Plant Cell Environ. 2021;44:1151–64 33522606 10.1111/pce.14010

[23] Machado KDC , Islam MT, Ali ES. et al. A systematic review on the neuroprotective perspectives of beta-caryophyllene. Phytother Res. 2018;32:2376–88 30281175 10.1002/ptr.6199

[24] Govindarajan M , Rajeswary M, Hoti SL. et al. Eugenol, α-pinene and β-caryophyllene from *Plectranthus barbatus* essential oil as eco-friendly larvicides against malaria, dengue and Japanese encephalitis mosquito vectors. Parasitol Res. 2016;115:807–15 26518773 10.1007/s00436-015-4809-0

[25] Dubey VS , Bhalla R, Luthra R. An overview of the nonmevalonate pathway for terpenoid biosynthesis in plants. Proc Anim Sci. 2003;28:637–46 10.1007/BF02703339 14517367

[26] Vranová E , Coman D, Gruissem W. Network analysis of the MVA and MEP pathways for isoprenoid synthesis. Annu Rev Plant Biol. 2013;64:665–700 23451776 10.1146/annurev-arplant-050312-120116

[27] Kitaoka N , Lu X, Yang B. et al. The application of synthetic biology to elucidation of plant mono-, sesqui-, and diterpenoid metabolism. Mol Plant. 2015;8:6–16 25578268 10.1016/j.molp.2014.12.002 PMC5120878

[28] Grotewold E . Transcription factors for predictive plant metabolic engineering: are we there yet? Curr Opin Biotech. 2008;19:138–44 18374558 10.1016/j.copbio.2008.02.002

[29] Chini A , Fonseca S, Fernández G. et al. The JAZ family of repressors is the missing link in jasmonate signaling. Nature. 2007;448:666–71 17637675 10.1038/nature06006

[30] Hong GJ , Xue XY, Mao YB. et al. *Arabidopsis* MYC2 interacts with DELLA proteins in regulating sesquiterpene synthase gene expression. Plant Cell. 2012;24:2635–48 22669881 10.1105/tpc.112.098749 PMC3406894

[31] Aslam MZ , Lin X, Li X. et al. Molecular cloning and functional characterization of CpMYC2 and CpBHLH13 transcription factors from wintersweet (*Chimonanthus praecox* L.). Plan Theory. 2020;9:785 10.3390/plants9060785 PMC7356763 32585874

[32] Xu W , Dubos C, Lepiniec L. Transcriptional control of flavonoid biosynthesis by MYB–bHLH–WDR complexes. Trends Plant Sci. 2015;20:176–85 25577424 10.1016/j.tplants.2014.12.001

[33] Li Y , Shan X, Gao R. et al. Two IIIf clade-bHLHs from *Freesia hybrida* play divergent roles in flavonoid biosynthesis and trichome formation when ectopically expressed in *Arabidopsis*. Sci Rep. 2016;6:30514 27465838 10.1038/srep30514 PMC4964595

[34] Łyczko J , Jałoszyński K, Surma M. et al. HS-SPME analysis of true lavender (*Lavandula angustifolia* mill.) leaves treated by various drying methods. Molecules. 2019;24:764 30791551 10.3390/molecules24040764 PMC6412978

[35] Wesołowska A , Jadczak P, Kulpa D. et al. Gas chromatography–mass spectrometry (GC–MS) analysis of essential oils from AgNPs and AuNPs elicited *Lavandula angustifolia* in vitro cultures. Molecules. 2019;24:606 30744099 10.3390/molecules24030606 PMC6385147

[36] Li J , Wang Y, Dong Y. et al. Correction: the chromosome-based lavender genome provides new insights into Lamiaceae evolution and terpenoid biosynthesis. Hortic. Res. 2021;8:90 33859175 10.1038/s41438-021-00536-9 PMC8050050

[37] Qiao DH , Tang MS, Jin L. et al. A monoterpene synthase gene cluster of tea plant (*Camellia sinensis*) potentially involved in constitutive and herbivore-induced terpene formation. Plant Physiol Bioch. 2022;184:1–13 10.1016/j.plaphy.2022.05.016 35613521

[38] Feng K , Kan XY, Yan YJ. et al. Identification and characterization of terpene synthase OjTPS1 involved in β-caryophyllene biosynthesis in *Oenanthe javanica* (Blume) DC. Ind Crop Prod. 2023;192:1–10

[39] Dong Y , Zhang W, Li J. et al. The transcription factor LaMYC4 from lavender regulates volatile terpenoid biosynthesis. BMC Plant Biol. 2022;22:289 35698036 10.1186/s12870-022-03660-3 PMC9190104

[40] Xu J , Van Herwijnen ZO, Dräger DB. et al. SlMYC1 regulates type VI glandular trichome formation and terpene biosynthesis in tomato glandular cells. Plant Cell. 2018;30:2988–3005 30518626 10.1105/tpc.18.00571 PMC6354261

[41] Yang Z , Li Y, Gao F. et al. MYB21 interacts with MYC2 to control the expression of terpene synthase genes in flowers of *Freesia hybrida* and *Arabidopsis thaliana* (R Hancock, Ed.). J Exp Bot. 2020;71:4140–58 32275056 10.1093/jxb/eraa184

[42] Lerdau M , Litvak M, Monson R. Plant chemical defense: monoterpenes and the growth-differentiation balance hypothesis. Trends Ecol Evol. 1994;9:58–61 21236767 10.1016/0169-5347(94)90269-0

[43] Tissier A , Morgan JA, Dudareva N. Plant volatiles: going ‘in’ but not ‘out’ of trichome cavities. Trends Plant Sci. 2017;22:930–8 28958712 10.1016/j.tplants.2017.09.001

[44] Dicke M , Sabelis MW, Takabayashi J. et al. Plant strategies of manipulating predatorprey interactions through allelochemicals: prospects for application in pest control. J Chem Ecol. 1990;16:3091–118 24263298 10.1007/BF00979614

[45] Kiefer IW , Slusarenko AJ. The pattern of systemic acquired resistance induction within the *Arabidopsis* rosette in relation to the pattern of translocation. Plant Physiol. 2003;132:840–7 12805614 10.1104/pp.103.021709 PMC167024

[46] Wenig M , Ghirardo A, Sales JH. et al. Systemic acquired resistance networks amplify airborne defense cues. Nat Commun. 2019;10:3813 31444353 10.1038/s41467-019-11798-2 PMC6707303

[47] Priya P , Yadav A, Chand J. et al. Terzyme: a tool for identification and analysis of the plant terpenome. Plant Methods. 2018;14:4 29339971 10.1186/s13007-017-0269-0 PMC5761147

[48] Khatri PK , Paolini M, Larcher R. et al. Botanical characterization and authentication of lavender essential oil using its volatile organic compounds and compound-specific carbon and hydrogen isotope ratio analysis. Food Control. 2023;154:1–11

[49] Tăbărasu AM , Anghelache DN, Găgeanu I. et al. Considerations on the use of active compounds obtained from lavender. Sustainability. 2023;15:8879

[50] Li H , Li J, Dong Y. et al. Time-series transcriptome provides insights into the gene regulation network involved in the volatile terpenoid metabolism during the flower development of lavender. BMC Plant Biol. 2019;19:313 31307374 10.1186/s12870-019-1908-6 PMC6632208

[51] Jin J , Kim MJ, Dhandapani S. et al. The floral transcriptome of ylang ylang (*Cananga odorata* var. fruticosa) uncovers biosynthetic pathways for volatile organic compounds and a multifunctional and novel sesquiterpene synthase. J Exp Bot. 2015;66:3959–75 25956881 10.1093/jxb/erv196 PMC4473991

[52] Matarese F , Cuzzola A, Scalabrelli G. et al. Expression of terpene synthase genes associated with the formation of volatiles in different organs of Vitis vinifera. Phytochemistry. 2014;105:12–24 25014656 10.1016/j.phytochem.2014.06.007

[53] Horsch RB , Fry JE, Hoffmann NL. et al. A simple and general method for transferring genes into plants. Science. 1985;227:1229–31 17757866 10.1126/science.227.4691.1229

[54] Clough SJ , Bent AF. Floral dip: a simplified method for *agrobacterium*-mediated transformation of *Arabidopsis thaliana*: floral dip transformation of *Arabidopsis*. Plant J. 1998;16:735–43 10069079 10.1046/j.1365-313x.1998.00343.x

[55] Jing T , Zhang N, Gao T. et al. Glucosylation of (Z)-3-hexenol informs intraspecies interactions in plants: a case study in *Camellia sinensis*. Plant Cell Environ. 2019;42:1352–67 30421786 10.1111/pce.13479

[56] Chen F , Tholl D, Bohlmann J. et al. The family of terpene synthases in plants: a mid-size family of genes for specialized metabolism that is highly diversified throughout the kingdom: terpene synthase family. Plant J. 2011;66:212–29 21443633 10.1111/j.1365-313X.2011.04520.x

[57] Kiran Babu GD , Sharma A, Singh B. Volatile composition of *Lavandula angustifolia* produced by different extraction techniques. J Essent Oil Res. 2016;28:489–500

[58] Jiang J , Xi H, Dai Z. et al. VvWRKY8 represses stilbene synthase genes through direct interaction with VvMYB14 to control resveratrol biosynthesis in grapevine. J Exp Bot. 2019;70:715–29 30445464 10.1093/jxb/ery401 PMC6322584

[59] He Z . Guidance to Experiment on Chemical Control in Crop Plants. Beijing: Beijing Agricultural University publication; 1993:

[60] Yang J , Zhang J, Wang Z. et al. Water deficit–induced senescence and its relationship to the remobilization of pre-stored carbon in wheat during grain filling. Agron J. 2001;93:196–206

[61] Chen T , Li Y, Xie L. et al. AaWRKY17, a positive regulator of artemisinin biosynthesis, is involved in resistance to *pseudomonas syringae* in *Artemisia annua*. Hortic Res. 2021;8:217 34593786 10.1038/s41438-021-00652-6 PMC8484609

