# Supplementary material for: LaMYC7, a positive regulator of linalool and caryophyllene biosynthesis, confers plant resistance to Pseudomonas syringae
Source: Hortic Res. 2024 Feb 6;11(4):uhae044. doi: 10.1093/hr/uhae044 (PMC11017519; doi:10.1093/hr/uhae044)
Supplement: Web_Material_uhae044 [file web_material_uhae044.zip › Fig S (0104).docx]

**Supplementary file**

**LaMYC7, a positive regulator of linalool and caryophyllene biosynthesis,** **confer plant resistance to *Pseudomonas syringae***

Yanmei Dong^1,2^, Ziling Wei^1,2,3^, Wenying Zhang^1,2,3^, Jingrui Li^1,2^, Meixian Han^1,2,3^, Hongtong Bai^1,2^, Hui Li^1,2*^, Lei Shi^1,2*^

^1^ State Key Laboratory of Plant Diversity and Specialty Crops, Institute of Botany, Chinese Academy of Sciences, Beijing 100093, China.

^2^ China National Botanical Garden, Beijing 100093, China.

^3^ University of Chinese Academy of Sciences, Beijing 100049, China.

* Correspondence: Lei Shi (shilei_67@126.com), Hui Li (lihui@ibcas.ac.cn)


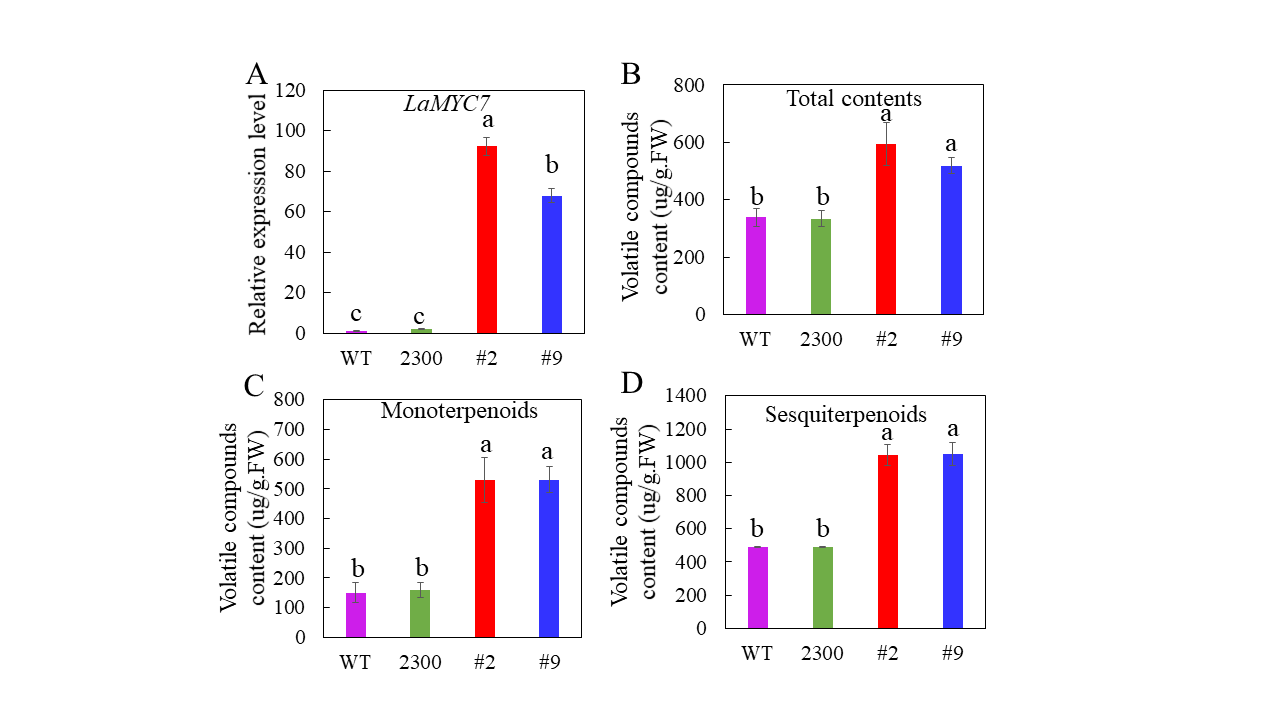


**Fig. S1** Analysis of VOCs from the tobacco floral. Wild type (WT), transformed by the empty vector pCAMBIA2300S (2300) and overexpressed LaMYC7 gene (35S::LaMYC7) plants (#2, #9). A, the relative expression level of *LaMYC7* was calculated by *NtTub* and *NtCYP* from tobacco floral. B-D, the contents of total contents, monoterpenoids and sesquiterpenoids. The products were identified by comparison with compounds in the library NIST14 and reference standards. Values shown are mean ± SD of three replicates. Standard errors are indicated as vertical lines on the top of each bar and bars annotated with different letters were significantly different according to Fisher’s LSD test (*P* < 0.05) after ANOVA.


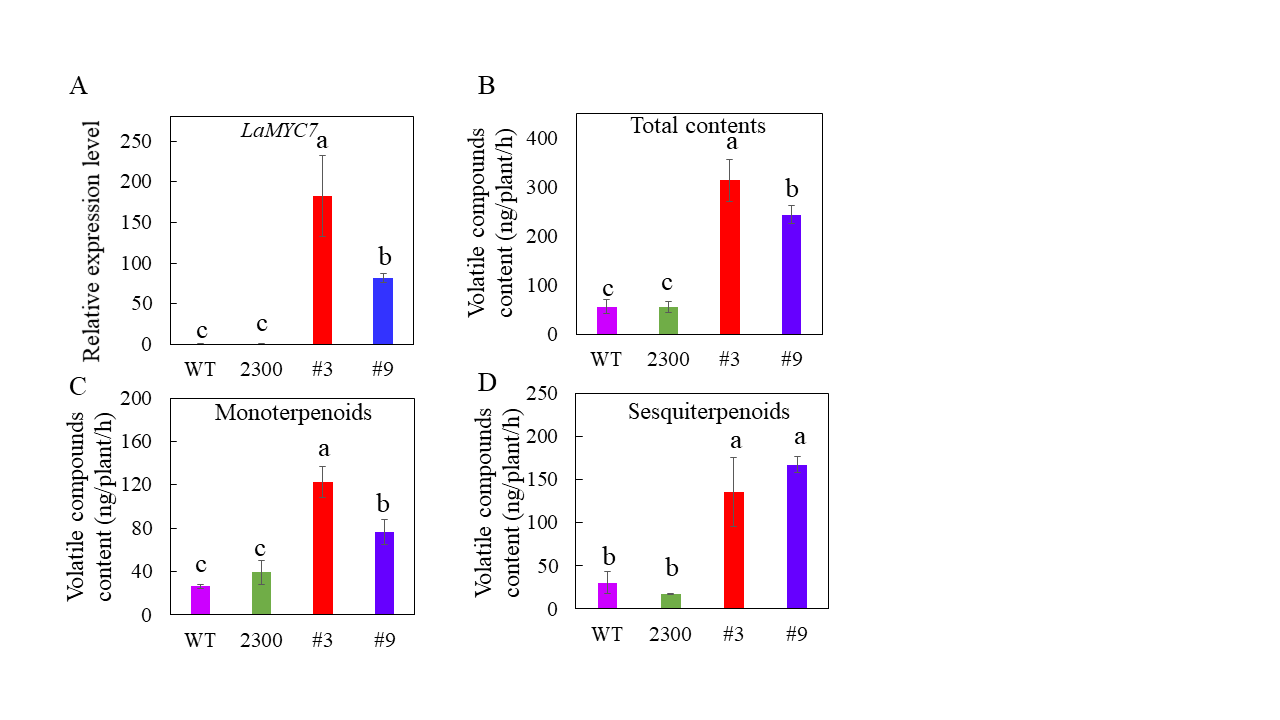


**Fig. S2** Analysis of VOCs from the Arabidopsis plants. Wild type (WT), transformed by the empty vector pCAMBIA2300S (2300) and overexpressed LaMYC7 gene (35S::LaMYC7) plants (#3, #9). A, the relative expression level of *LaMYC7* was calculated by *AtTubulin* and *Atβ-actin* from *Arabidopsis* floral. B-D, the contents of total contents, monoterpenoids and sesquiterpenoids. The products were identified by comparison with compounds in the library NIST14 and reference standards. Values shown are mean ± SD of three replicates. Standard errors are indicated as vertical lines on the top of each bar and bars annotated with different letters were significantly different according to Fisher’s LSD test (*P* < 0.05) after ANOVA.


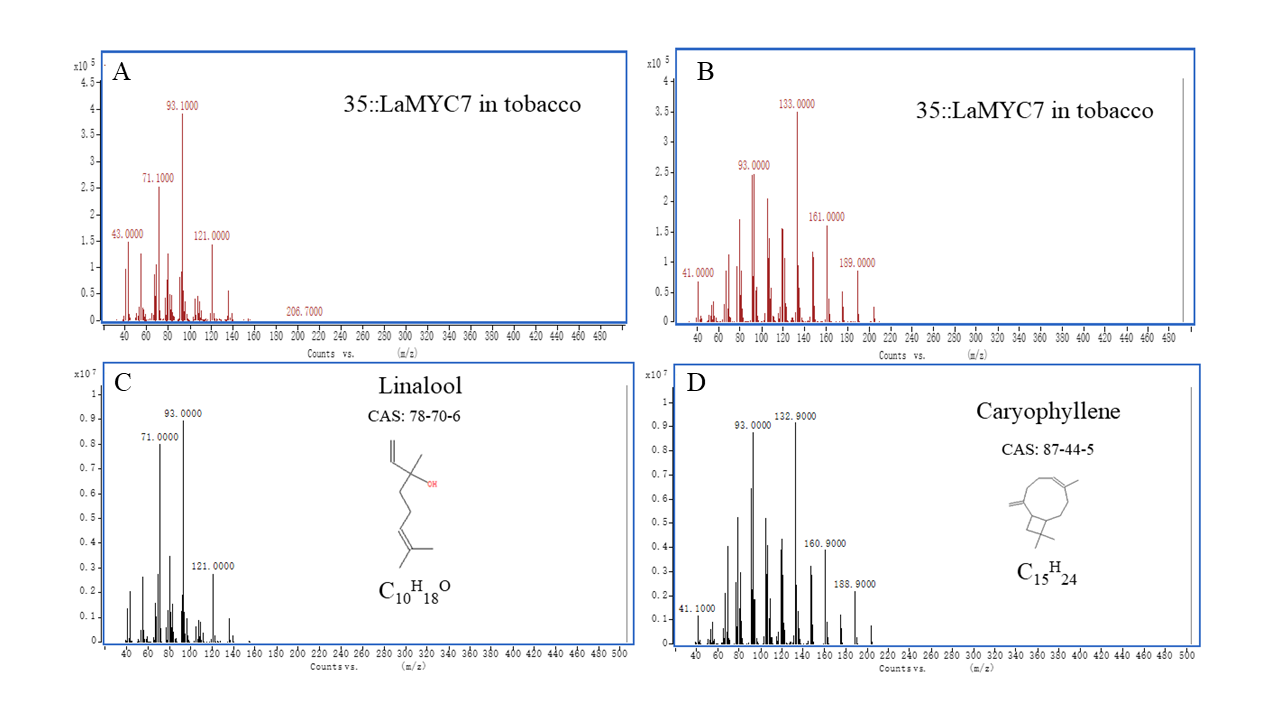


**Fig. S3** Mass spectrum of linalool and caryophyllene. A, B, Mass spectrum of the product in tobacco. C, D, Mass spectrum of linalool and caryophyllene.


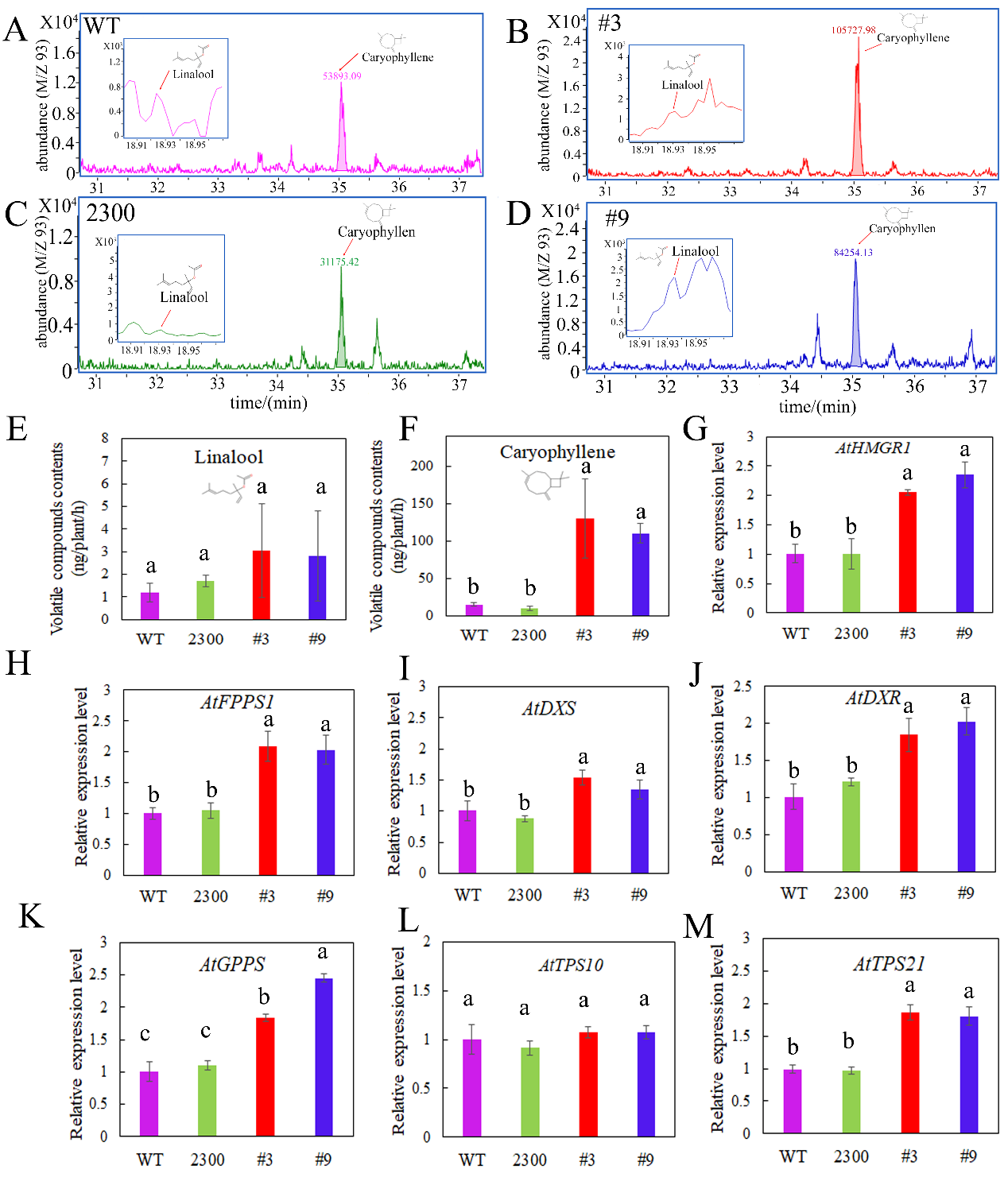


**Fig. S4** Analysis of the *LaMYC7*-overexpressing in *A. thaliana*. Wild-type (WT), plants transformed with the empty vector pCAMBIA2300 (2300) and *LaMYC7-* overexpressing plants with 35S::LaMYC7 (#3, #9). (A-D) GC trace of caryophyllene and linalool. The peak area was indicated by the number on the peak. (E) Linalool content. (F) Caryophyllene content. (G-M) Relative expression levels of *AtHMGRL*, *AtFPPS1*, *AtDXS*, *AtDXR*, *AtGPPS*, *AtTPS10* and *AtTPS21*. By comparing the products to substances in the NIST14 collection and reference standards, the compounds were identified. The numbers displayed are the average of at least three replicates (mean ± SD). Following an ANOVA, Fisher's LSD test revealed that bars labeled with various letters were significantly different (*P* < 0.05), as seen by the vertical lines at the top of each bar indicating standard errors.


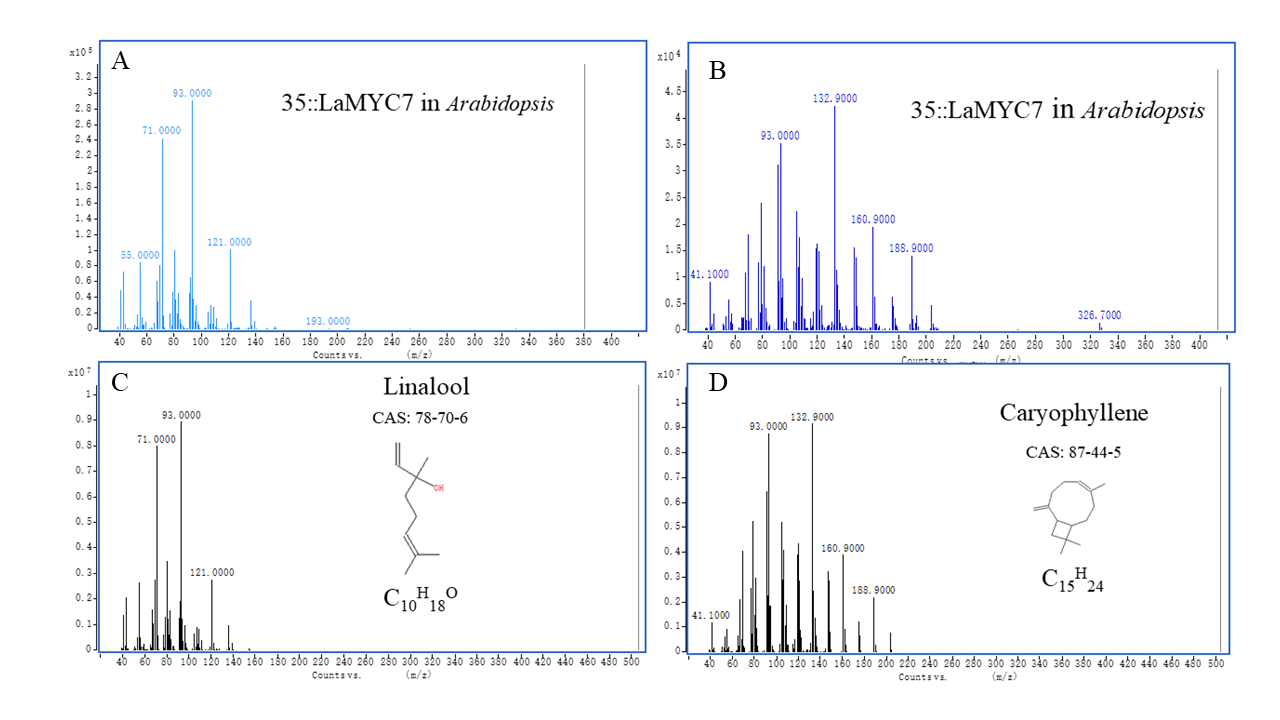


**Fig. S5** Mass spectrum of linalool and caryophyllene. A, B, Mass spectrum of the product in *Arabidopsis*. C, D, Mass spectrum of linalool and caryophyllene.


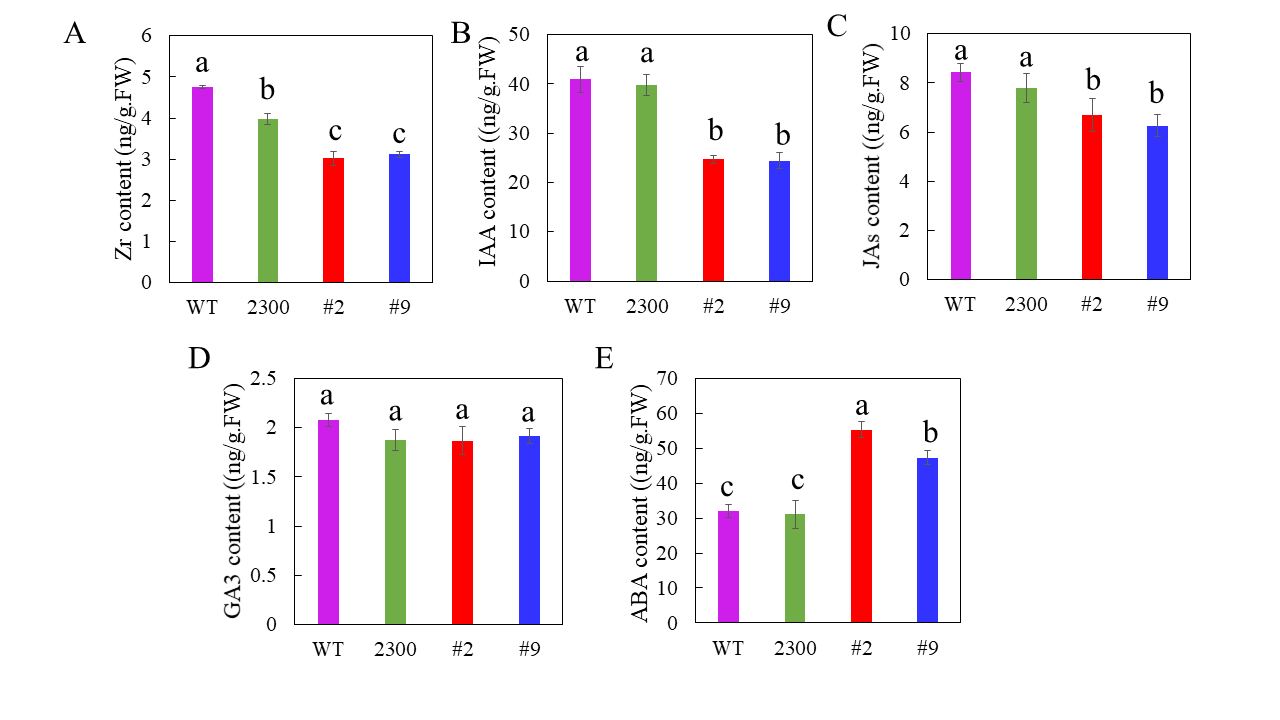


**Fig. S6** Hormone contents from the tobacco leaves. Hormone contents were measures by Enzyme-linked immunosorbent assay (ELISA). WT, wild type; 2300, transformed by the empty vector pCAMBIA2300S; #2 and #9, LaMYC7 transgenic lines. Values shown are mean ± SD of three replicates. Standard errors are indicated as vertical lines on the top of each bar and bars annotated with different letters were significantly different according to Fisher’s LSD test (*P* < 0.05) after ANOVA.


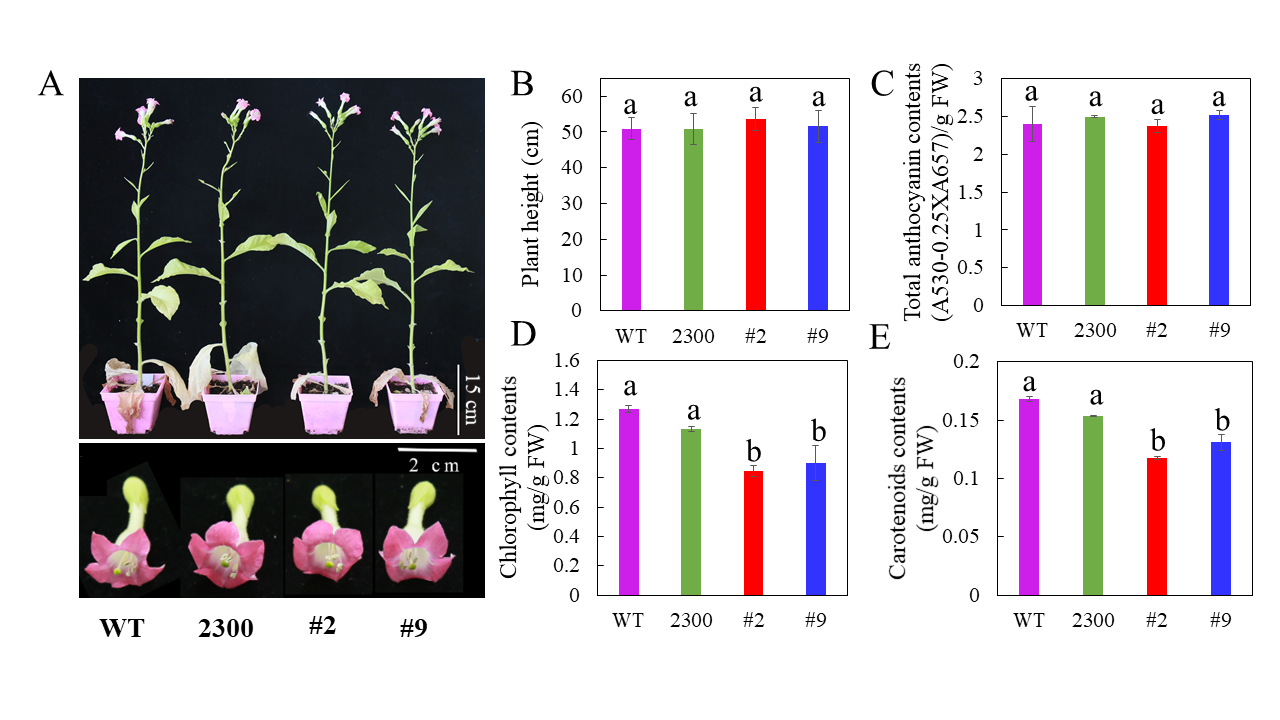


**Fig. S7** Phenotypic analysis of LaMYC7 transgenic tobacco. A, Phenotypes of plant and flowers in wild type (WT), transformed by the empty vector pCAMBIA2300S (2300) and LaMYC7 transgenic lines (#2, #9). B, Results of plant height. C, Total anthocyanin content in tobacco flowers. D, Chlorophyll contents. E, Carotenoids contents. Values shown are mean ± SD at least three replicates. Standard errors are indicated as vertical lines on the top of each bar and bars annotated with different letters were significantly different according to Fisher’s LSD test (*P* < 0.05) after ANOVA.


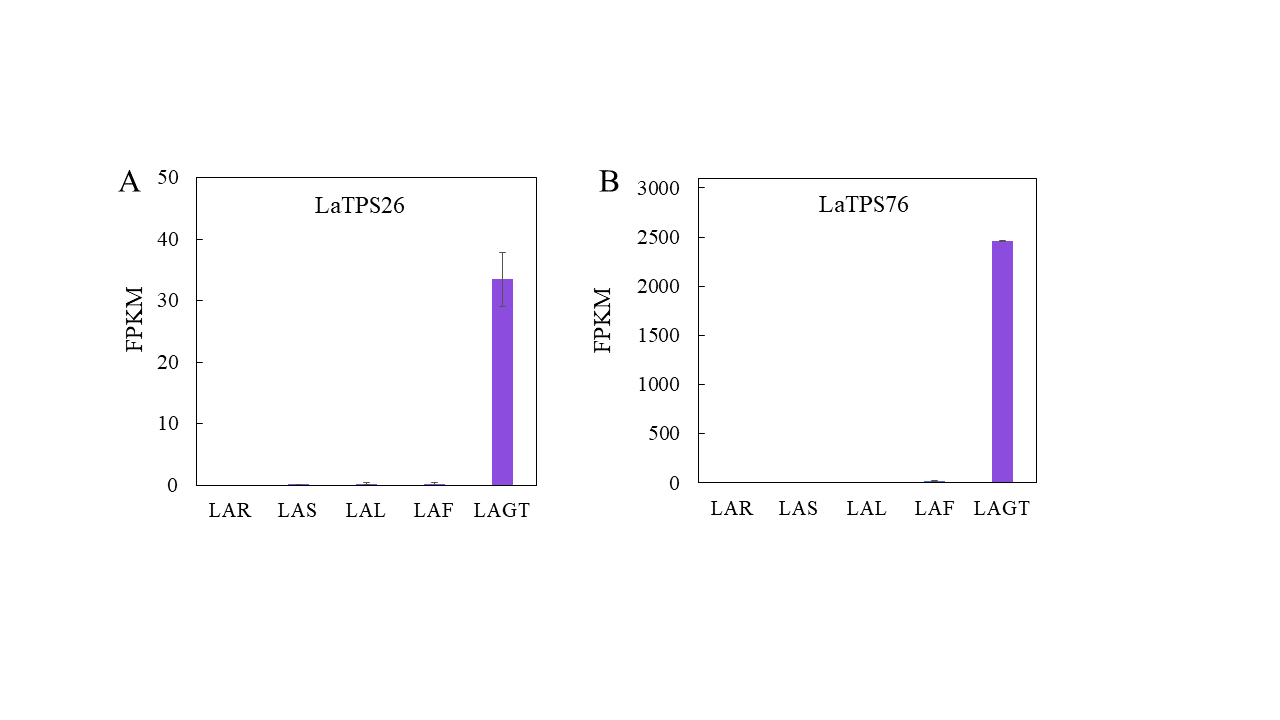


**Fig. S8** Transcriptional abundance of *LaMYC7* in different tissues (LAR, root; LAS, stem; LAL, leaf; LAF, flower; LAGT, glandular trichome).


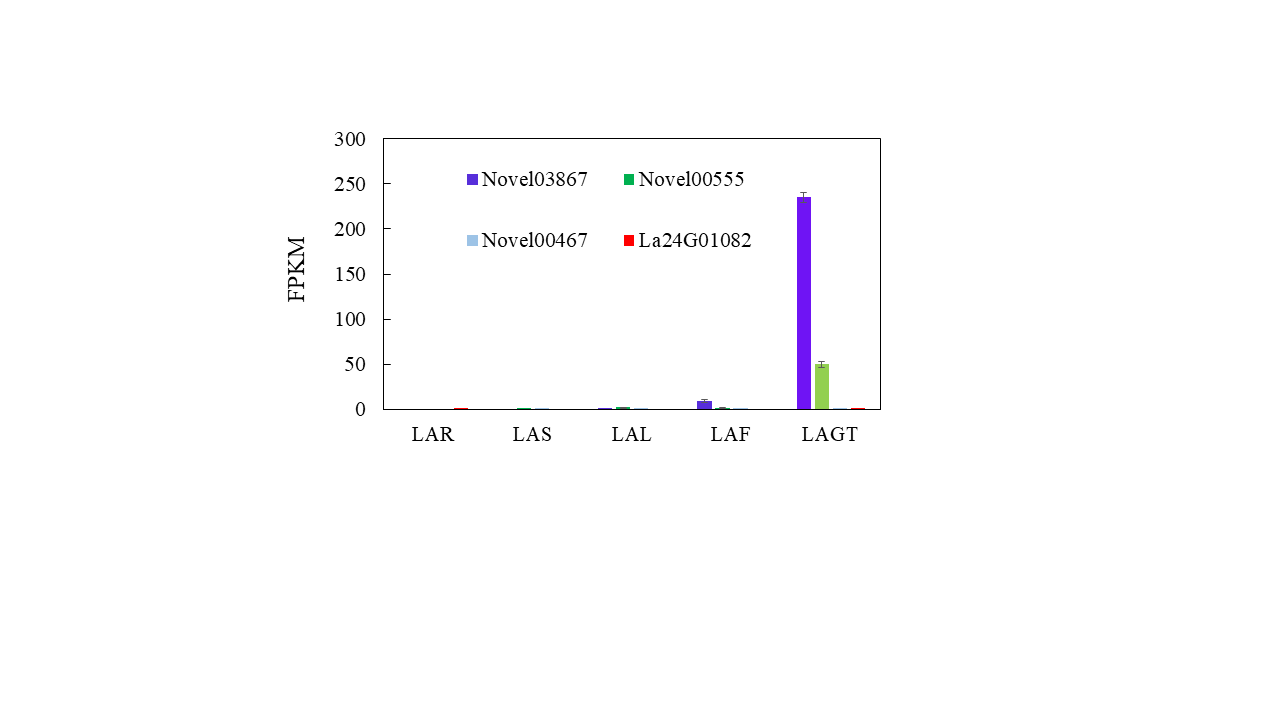


**Fig. S9** Transcriptional abundance of four homologous genes of linalool synthase in different tissues (LAR, root; LAS, stem; LAL, leaf; LAF, flower; LAGT, glandular trichome).


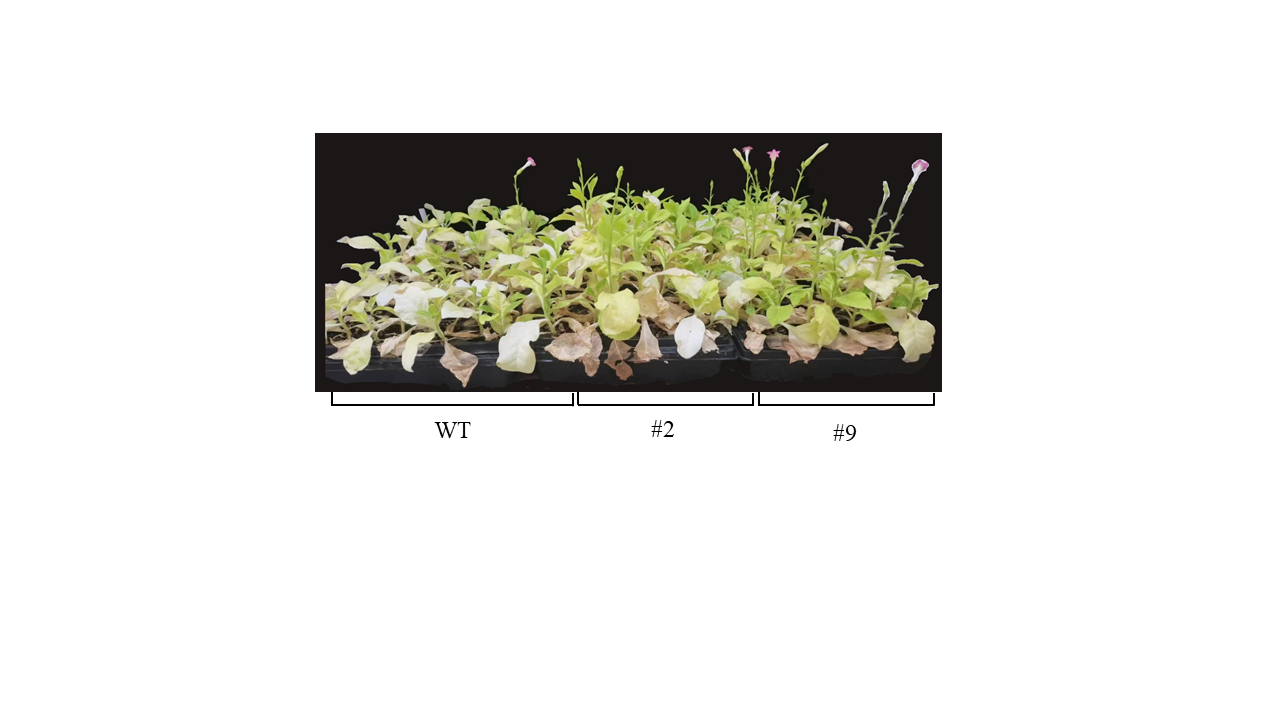


**Fig. S10** Phenotypic analysis of LaMYC7 transgenic tobacco in drought treatment. WT, wild type; #2 and #9, LaMYC7 transgenic lines.
